# The novel sterilization device: the prototype testing

**DOI:** 10.1038/s41598-023-44483-y

**Published:** 2023-10-13

**Authors:** Robert Nowak, Paweł Wityk, Anna Wierzbicka-Woś, Waldemar Gos, Dorota Kostrzewa-Nowak

**Affiliations:** 1 Institute of Physical Culture Sciences, University of Szczecin, 17C Narutowicza St., 70-240 Szczecin, Poland; 2 Department of Pathology, Pomeranian Medical University in Szczecin, 1 Unii Lubelskiej St., 71-242 Szczecin, Poland; 3 Faculty of Chemistry, Gdańsk University of Technology, 11/12 Narutowicza St., 80-233 Gdańsk, Poland; 4 Department of Biopharmaceutics and Pharmacodynamics, Faculty of Pharmacy, Medical University of Gdańsk, 107 Hallera St., 80-416 Gdańsk, Poland; 5 The Centre for Molecular Biology and Biotechnology, University of Szczecin, 13 Wąska St., 71-415 Szczecin, Poland; 6 Present Address: Research and Development Centre, Sanprobi Sp. z o. o. Sp. k., 5/c Kurza stopka St., 70-535 Szczecin, Poland; 7 Institute of Economics and Finance, University of Szczecin, 64 Mickiewicza St., 71-101 Szczecin, Poland; 8 Department of Clinical and Molecular Biochemistry, Pomeranian Medical University in Szczecin, 72 Powstańców Wlkp. Al., 70-111 Szczecin, Poland

**Keywords:** Health care, Microbiology

## Abstract

Currently, there are numerous methods that can be used to neutralize pathogens (i.e., devices, tools, or protective clothing), but the sterilizing agent must be selected so that it does not damage or change the properties of the material to which it is applied. Dry sterilization with hydrogen peroxide gas (VHP) in combination with UV-C radiation is well described and effective method of sterilization. This paper presents the design, construction, and analysis of a novel model of sterilization device. Verification of the sterilization process was performed, using classical microbiological methods and flow cytometry, on samples containing *Geobacillus stearothermophilus* spores, *Bacillus subtilis* spores, *Escherichia coli*, and *Candida albicans*. Flow cytometry results were in line with the standardized microbiological tests and confirmed the effectiveness of the sterilization process. It was also determined that mobile sterilization stations represent a valuable solution when dedicated to public institutions and businesses in the tourism sector, sports & fitness industry, or other types of services, e.g., cosmetic services. A key feature of this solution is the ability to adapt the device within specific constraints to the user’s needs.

## Introduction

The statistical data clearly shows that the sterilization market has grown significantly in 2019 compared with 2018; in 2020, it is likely to contract following a collapse of the supply chain, the introduction of other, more effective products to the market, or a reduced industrial demand for sterilization services. The global COVID-19 pandemic triggered the need for increased use of decontamination and sterilization tools^1–3^, which induced growth in the global sterilization market^4,5^. Economic analysis indicated that high-temperature sterilization processes are the most important methods available, whereas processes that use low temperatures correspond to a much smaller share of the market (e.g., sterilization with H_2_O_2_). It is expected that this situation will further promote the growth of the sterilization services market in the coming years^4,5^. However, concerns regarding the safety of re-used tools^6–8^ are expected to limit market growth to some extent in the coming years. Considering end-user non-compliance with sterilization standards, this is expected to limit the growth of this market. These factors have inspired the search for a native production technology for mobile sterilization devices.

Many methods can be applied to eliminate pathogens. However, the choice of sterilizing agent depends on the type of sterilized material because the base material cannot be damaged and must retain its properties^8–10^. Sterilization of numerous types of materials can be achieved through dry sterilization techniques with hydrogen peroxide gas (H_2_O_2_) and UV-C irradiation. Relative to other techniques for neutralizing pathogens (e.g., autoclaving, sterilization with ethylene oxide, X-rays, or gamma rays), this approach is safer, faster, and cheaper, and it does not damage the sterilized materials by using high temperatures^6–8,11,12^. A mobile sterilization station represents a solution dedicated to public institutions and businesses in key industries (e.g., tourism, sports and fitness, cosmetic services, etc.). Moreover, the device could be adapted to the user’s specific needs. Hence, the aim of the present work was to design and construct a mobile sterilization station comprising a device that enables the sterilization of personal protective equipment, office equipment, teaching aids, and sports gear, among other elements that are used repeatedly in places experiencing increased demand (e.g., in stationary units and field service points of uniformed services: police, fire brigades, municipal police, etc.). A device that can simultaneously, rapidly, and precisely sterilize numerous surfaces without the need for specialized personnel, and without incurring the high costs associated with purchasing special devices and neutralizing the waste generated. The vaporized hydrogen peroxide and UV-C to sterilize reusable metal and nonmetal devices and it has limitations, mainly related with lower penetration capabilities. This technique is compatible with a wide range of materials in common use (e.g., polypropylene, brass, polyethylene) but is not intended to process liquids, linens, powders, or any cellulose or nylon materials. The system can sterilize instruments with diffusion-restricted spaces (e.g., scissors) and a single stainless-steel lumen based on lumen internal diameter and length (e.g., an inside diameter of 1 mm or larger and a length of 125 mm or shorter^13,14^.

This work describes the design, construction, and evaluation of a novel mobile sterilization device. The research included the development and preparation of a working prototype of a mobile sterilization station, to assess its competitiveness in the field of clothing and personal protective material sterilization. Our proprietary solution involves the use of commonly available materials, which improves access to the sterilization equipment for non-specialized personnel. Furthermore, it is based on simple and user-safe sterilization factors that do not generate environmentally hazardous waste. The fundamental research, as well as research development activities (completed at the technology readiness level 8; TLR8) included (1) planning and constructing a prototype that generates dry H_2_O_2_ vapors for sterilization, and (2) microbiological verification and optimization of the sterilization process using the prototype (sterilization parameter optimizations in terms of temperature, humidity, and treatment time), while focusing on the real-life conditions.

## Results

### Microbiological verification and optimization of the sterilization process using the prototype

The first tests focused on optimizing the duration and temperature of the sterilization process. During these tests, various concentrations of H_2_O_2_ and a range of temperatures were applied during the sterilization process (Table 1).

**Table 1:** The effect of hydrogen peroxide vapors on *B. subtilis* spores and *E. coli* cells placed on cellulose.

Microorganisms	Dry H_2_O_2_ vapor concentration (ppm)	Time of the exposure to dry H_2_O_2_ vapors			
		0 min (K−)	15 min	20 min	30 min
*B. subtilis* spores placed on cellulose strips containing 2 × 10^6^ (CFUs)	OD_600_ values depending on the time of exposure to the sterilizing agent at 25–30 °C				
	50	2.1	1.0	0.5	0.0
	100	2.0	0.0	0.0	0.0
	150	2.0	0.0	0.0	0.0
	OD_600_ values depending on the time of exposure to the sterilizing agent at 35–40 °C				
	50	2.2	0.0	0.0	0.0
	100	2.1	0.0	0.0	0.0
	150	2.2	0.0	0.0	0.0
*E. coli* cells placed on cellulose strips containing 2 × 10^7^ CFUs	OD_600_ values depending on the time of exposure to the sterilizing agent at 25–30 °C				
	50	0.025	0.02	0.01	0.00
	100	0.024	0.00	0.00	0.00
	150	0.023	0.00	0.00	0.00
	OD_600_ values depending on the time of exposure to the sterilizing agent at 35–40 °C				
	50	0.021	0.00	0.00	0.00
	100	0.022	0.00	0.00	0.00
	150	0.023	0.00	0.00	0.00

OD_600_—optical density of a microbial culture at a wavelength of 600 nm, correlated with the number of microbial cells; mean OD_600_ values determined after the scheduled incubation time of the test strips in TSB liquid medium; (K−)—negative control (test strip not exposed to the sterilization agent).

**Figure 1:**
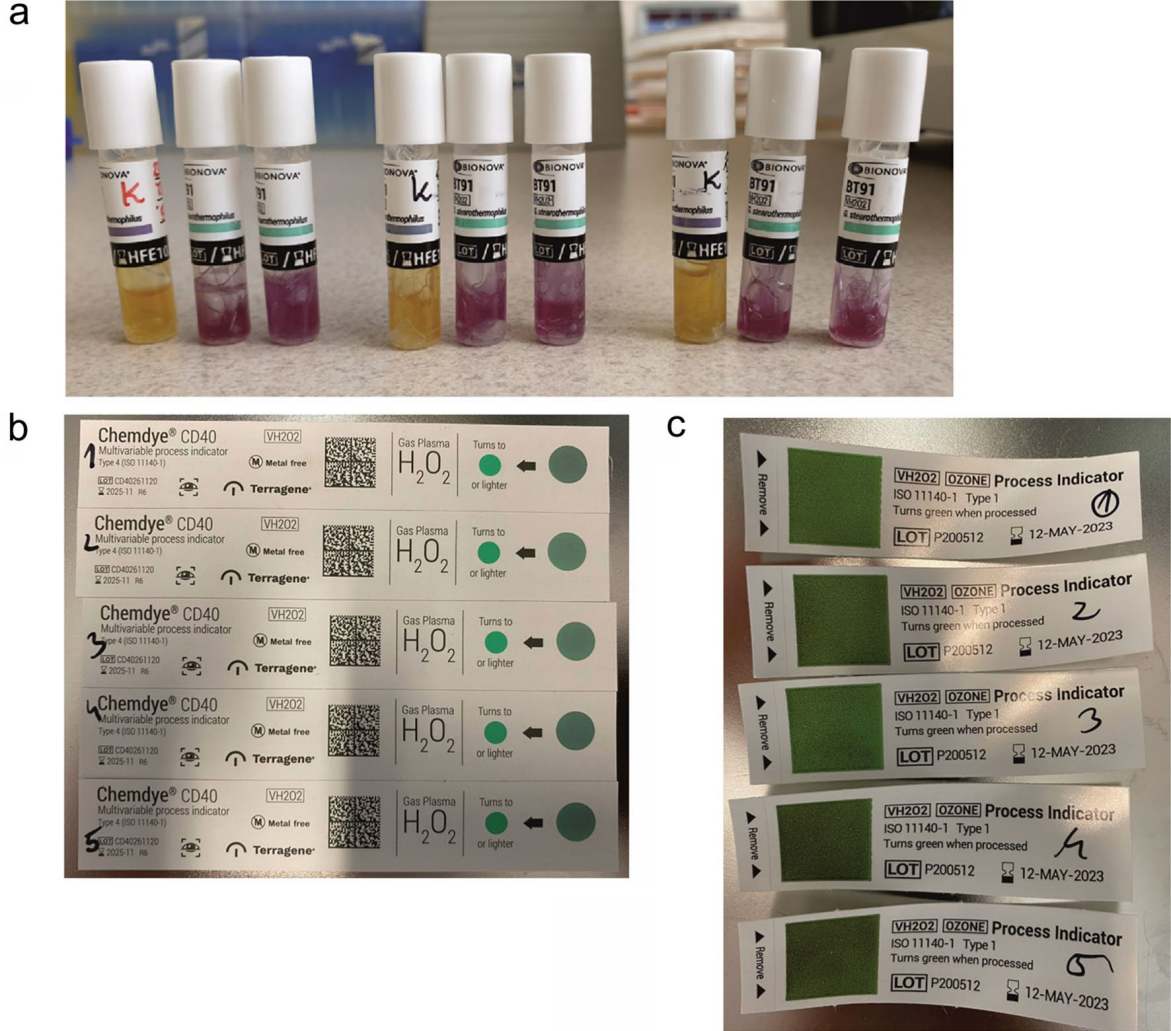
Quality controls used in the study. (**a**) Standardized biological quality controls containing *G. stearothermophilus* (ATCC7953) spores with CFU 1.3 × 10^5^ and a liquid culture medium with a color indicator (additionally equipped with chemical strips to indicating exposure to H_2_O_2_ vapors). Vials that were not sterilized are labelled with “K” (yellow medium indicates bacterial growth) and vials after completing the sterilization process show a violet color (indicating a lack of bacterial growth). (**b**) Chemdye CD40 type-4 commercial chemical tests strips confirming the effectiveness of the H_2_O_2_ vaporization (test field colored green) in the tested sterilization station. (**c**) VH2O2 Process Indicator strips type-1 confirming the effectiveness of the H_2_O_2_ vaporization (test field colored green) in the tested sterilization station.

On the basis of those preliminary results, 150 ppm of dry H_2_O_2_ vapors, a temperature of 35–40 °C, and a treatment duration of 20 min were selected for further tests. In the first step of the decontamination process, 5 min of UV-C irradiation was applied. The UV-C irradiation was also applied at the end of the sterilization process to promote the chemical decomposition of dry H_2_O_2_ vapors. Therefore, the total UV-C radiation dose was equal to 2.76 J/cm^2^. The microbiological quality control involved commercial standardized biological test with indicator bacterial strain spores *Geobacillus stearothermophilus* being compliant with ISO 11138-1^15^ standard (Fig. 1a) and often occurring in the human environment microbial strains represents bacteria gram-positive, gram-negative and yeasts such as *B. subtilis, E. coli* and *C. albicans*, respectively. Simultaneously, the effectiveness of H_2_O_2_ vapors for the sterilization was monitored using two types of commercially available chemical tests: Chemdye CD40 type-4 (Fig. 1b) and VH2O2 Process Indicator type-1 compliant with ISO 11140-1 standard^16^ (Fig. 1c). Tested microorganisms exposed on various conditions in this study confirmed that the mobile sterilization station effectively destroyed bacterial and yeast cells on used materials (Table 1). The percentage of dead vs live cells was also analyzed by flow cytometry to determine the best parameters for sterilization process which leads to over 99.9% of inactivated microbial cells (Table 2).

**Table 2:** Percentage of dead *B. subtilis* and *E. coli* cells after exposure to hydrogen peroxide at 35–40 °C analyzed by flow cytometry.

Dry H_2_O_2_ vapor concentration (ppm)	0 min (K−) of exposure	15 min of exposure	20 min of exposure	30 min of exposure
Percentage of dead *B. subtilis* cells				
50	2.0	91.1	93.4	94.6
100	1.8	98.2	98.8	99.2
150	2.1	99.1	99.7	99.9
Percentage of dead *E. coli* cells				
50	0.73	96.5	99.7	99.9
100	0.88	98.2	99.9	99.9
150	0.86	99.6	99.9	100

(K−)—negative control (test strip not exposed to sterilization agent).

**Figure 2:**
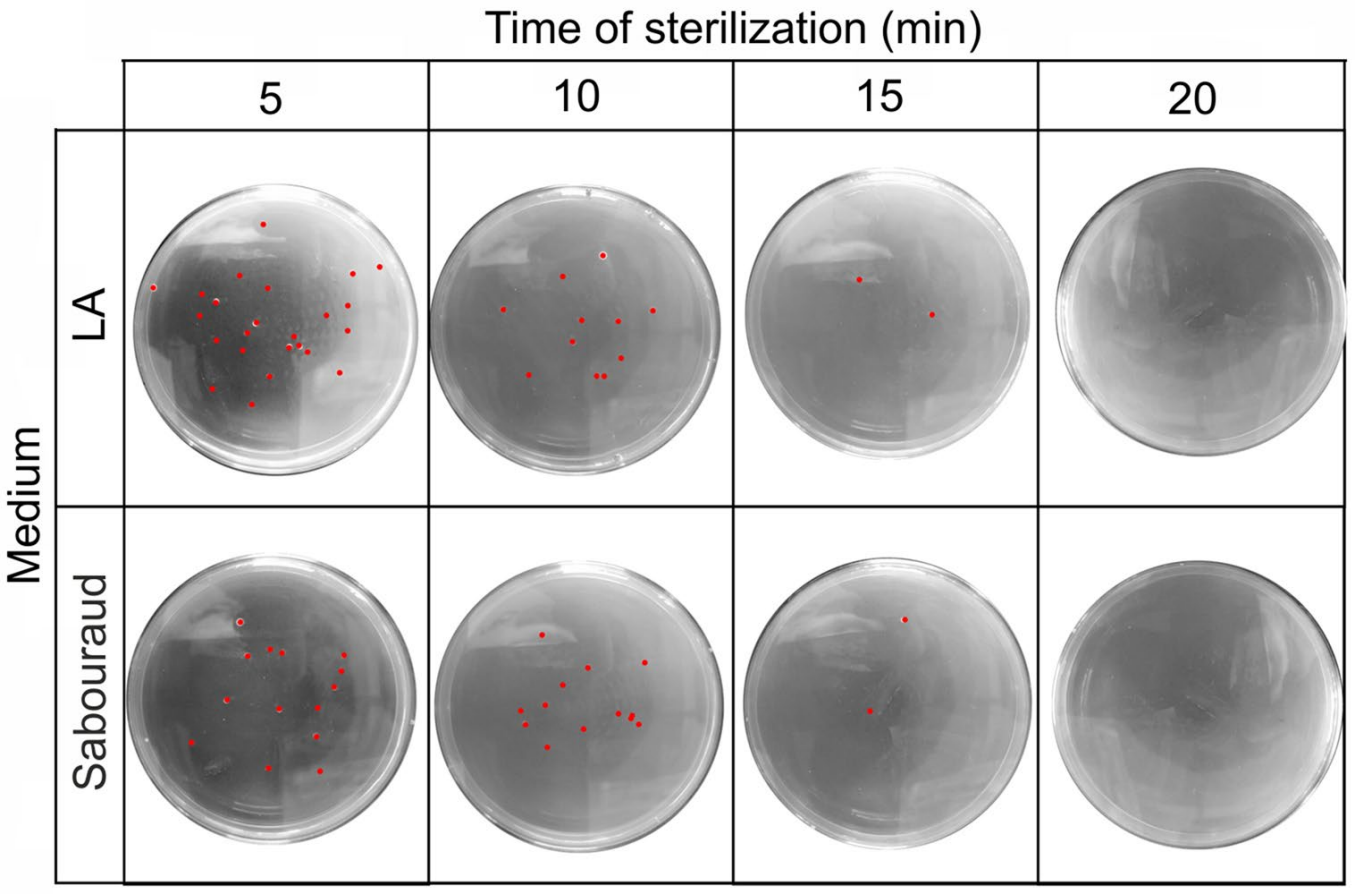
Representative photographs of microbial cultures on solid medium after the sterilization process. Red dots represent colony forming units (pseudo-colored).

The second set of biological performance tests evaluated the mobile sterilization station’s ability to sterilize the surface of materials and devices placed in the working chamber as well as the air from the working chamber (using a MicroBio MB1 microbiological air sampler) during full sterilization cycles. Results shown that after 20 min of sterilization (150 ppm of dry H_2_O_2_ vapors, at 35–40 °C) no microorganisms’ growth was observed on culture media (Table 3, Fig. 2).

**Table 3:** Number of colonies on LA and Sabouraud media (after incubation at 37 °C for 48 h and subjecting to a stream of 10 L of air from the working chamber following 5–20 min of sterilization).

Number of colonies					
Time (min)	0	5	10	15	20
LA medium	94	24	7	1	0
	115	19	8	2	0
Sabouraud medium	87	12	9	1	0
	91	17	11	2	0
Mean ± SD	97 ± 11	18 ± 4	9 ± 1	2 ± 1	0 ± 0
Calculated CFUs/m^3^	9675 ± 1083	1800 ± 430	875 ± 148	150 ± 50	0 ± 0

**Figure 3:**
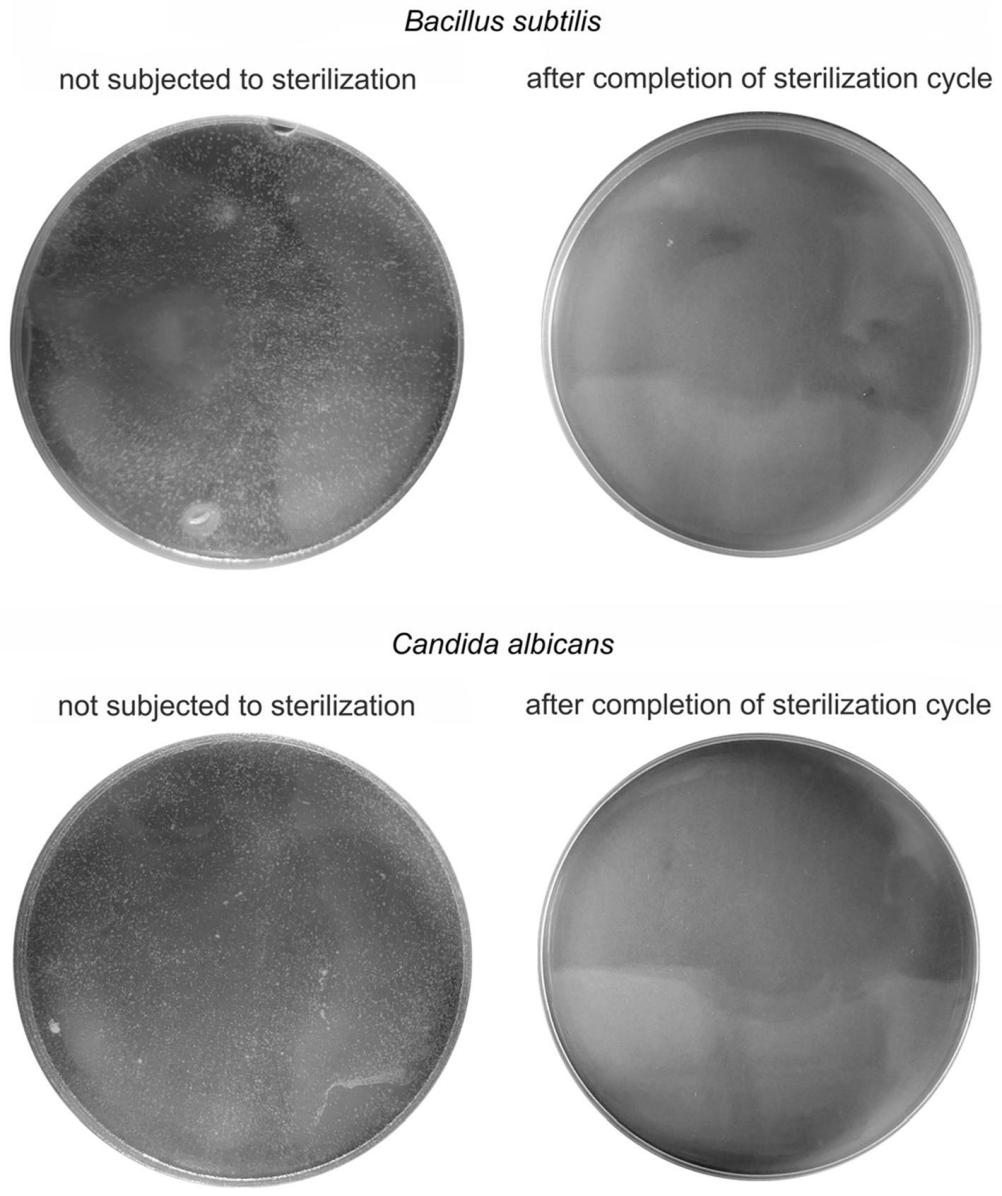
Representative results of cultures on a solid medium; microorganisms were washed off of the surface of discs before and after the sterilization process. After completing a sterilization cycle, the microorganisms were rinsed from the discs with PBS and plated on LA or Sabouraud Petri dishes.

The sterilization of cellulose-cotton materials using the mobile sterilization station was verified by testing discs (diameter = 1 cm, thickness = 2–4 mm) soaked with the selected microorganisms. The discs were placed in the device and sterilized using dry H_2_O_2_ vapor at a concentration of 150 ± 5 ppm, under a total UV-C irradiation dose of 2.76 J/cm^2^, at a temperature of 38 °C. After completing a sterilization cycle, the microorganisms were rinsed from the discs with phosphate-buffered saline (PBS) and plated on non-selective Luria Bertani agar (LA) or Sabouraud agar media on Petri dishes. After incubation no microorganisms’ growth was observed on plates with samples after completing the sterilization process, in contrast to control plates with samples without sterilization, where lawn-growth of microorganisms was observed (Fig. 3). The results for the control (discs not subjected to the sterilization process) are presented in Fig. 3.

### Focused research

An important element of prototyping involved testing the developed sterilization station in real-life conditions (with volunteer participants).

For this purpose, the mobile sterilization station operation was demonstrated for volunteer participants who constituted focus group. The study was conducted based on a proprietary questionnaire to assess the device according to user evaluations and to confirm the effectiveness of the microbiological sterilization of everyday items. Office items (school rulers, set squares, pens, pencils, keys) and personal protective equipment (masks, helmets), were tested for this research. Participants from focus group who performed at least several cycles of sterilization process (operated the sterilization station) were asked to fill in the questionnaire form. The user survey was completed by 46 respondents, 22% of whom were women; 26 participants had completed some degree of higher education, two had completed primary education, and the remainder had a secondary education level; the median age of participants was 35 years (ranging from 22 to 57 years); 70% of respondents agreed that there is a need to use mobile sterilization stations, and over 75% believed that this solution would increase work safety. The overall assessment of the sterilizing unit was good (about 62%). The quality of workmanship, aesthetics of the device, ease of use, intuitiveness, and readability were also rated very highly. The worst-rated parameters were ease of spare part replacement and ease of transport. According to the respondents, the distinguishing features of the mobile sterilization station were: “ease of use”, “quality of sterilization, sterilization time, and sterilization efficiency”, and “the possibility of sterilizing teaching materials”.

### Testing under real conditions

The mobile sterilization station was tested under real conditions by members of the state fire brigade, who used the station for three months, while noting any comments and observations. After the testing period they provided feedback indicating that this equipment was very useful from their perspective. The three months of testing confirmed that the construction (materials used to build the machines and interface used to operate them) was adequate to satisfy the users’ needs. It also indicated that the maintenance of the station is facile and not time consuming, and there was no damage or failure that reduced the usability of the device. Moreover, the sterilization process is relatively short, and therefore, the amount of wasted time is minimal.

The microbiological controls (standardized biological quality controls containing *G. stearothermophilus*) verified the effectiveness of the sterilization process at the end of the test. It is worth noting that the mobile sterilization stations were transported between different users. Importantly, the device transport did not affect its sterilization efficiency, and the preparation for transport and device restart were easy for the users. Following our experiments, all prototype models of the mobile sterilization station were donated to the fire brigade station in Western Pomerania (Poland) and are still in use during the pandemic. At the time of writing, there have been no indications of any damages or device failures.

## Discussion

Hydrogen peroxide is commonly used as an antimicrobial agent; it decomposes rapidly to water and oxygen, which are nontoxic, and it is therefore safe and effective to use for biologic deactivation purposes^11^. Vaporized hydrogen peroxide (VHP) is a form of hydrogen peroxide that exhibits effective lethality against a wide range of microorganisms while remaining nontoxic to human health^17^. VHP seems to be a more effective disinfectant than 0.5% sodium hypochlorite solution at eradicating *Clostridium difficile* spores and is recommended as a novel alternative for disinfecting patients’ rooms^18,19^. It also helps to eliminate hospital infections caused by methicillin-resistant *Staphylococcus aureus* (MRSA)^20^. Verification of the usefulness of VHP at low concertation in our novel sterilization station was performed using microbiological tests on standardized biological quality control samples containing *Geobacillus stearothermophilus, Bacillus subtilis* spores, *Escherichia coli,* and *Candida albicans*. We confirmed the effectiveness of the sterilization process against selected microbial pathogens at a VHP concentration of 150 ppm. These microbiological tests were performed using both classical microbiological methods and flow cytometry, with the results of the flow cytometry analyses in line with the standardized microbiological tests. The VHP system is a relatively quick and user-friendly technology, and for this reason it was used in the prototype mobile sterilization station. However, it was evident that the VHP residue left in the sterilization chamber at the end of the process was chemical waste that is a danger to the user. Applying UV-C irradiation is also an effective method for converting H_2_O_2_ into nontoxic derivatives. In fact, the UV-C irradiation also might be the main sterilization agent against *Bacillus* and fungal spores sprayed onto substrates, as described by Halfmann et al.^10,21^. It is worth noting that VHP disinfection leads to complete inactivation of some viruses, including poliovirus, rotavirus, adenovirus, murine norovirus^22^, and SARS-CoV-2^23^, although an extended cycle time was required relative to Indiana serotype (VSV)^7^. Moreover, a pioneering study conducted by Criscuolo et al.^12^ demonstrated the deactivation of SARS-CoV-2 on different materials under UV-C irradiation and ozone exposure. However, the use of our prototype for inactivation of other viruses requires future evaluation.

Our prototype was effective against the bacteria analyzed and *C. albicans,* and it is easy to construct and use by non-specially trained users. This prototype is a solution to the increasing global need for sterilization-dedicated equipment for non-medical units. The developed sterilization station combines multiple decontamination and sterilization factors, namely UV-C irradiation and VHP. Microbiological tests conducted under laboratory and real-life conditions confirmed that this device is effective against microbes that are commonly used to validate sterilization techniques. Overall, this study demonstrated that the mobile sterilization station can be used to sterilize small everyday items, as well as homemade masks. It must be pointed out that the device was projected mainly for items which do not necessarily have to be sterile, but need to be decontaminated after contact with a potential infectious agent that the staff may have come into contact with earlier. Therefore, recommendations and directions for use quality control (commercially available chemical tests are sufficient) are included in the user manual provided with the device.

The present report further indicates that the developed mobile sterilization station is not only an effective sterilization tool, but it can also fill the market demand for new adaptable technological solutions.

## Conclusions

Advancements in the sterilization of clothing and implementation of these solutions (device prototypes) will enable the wide application of novel techniques in the field for various industries (e.g., tourism, sport and fitness, beauty and wellness) and special services (e.g., fire brigade, army, police). The system developed herein can be adapted to the needs of the user (e.g., by streamlining the process, simplifying its operation, developing different versions of the device depending on the needs of the end user), and it could even be developed as an intelligent device when operating based on remote controls. The presented mobile sterilization station received a hygienic certificate and is ready to be Conformité Européenne (CE) certified.

## Materials and methods

### Prototype construction

**Figure 4:**
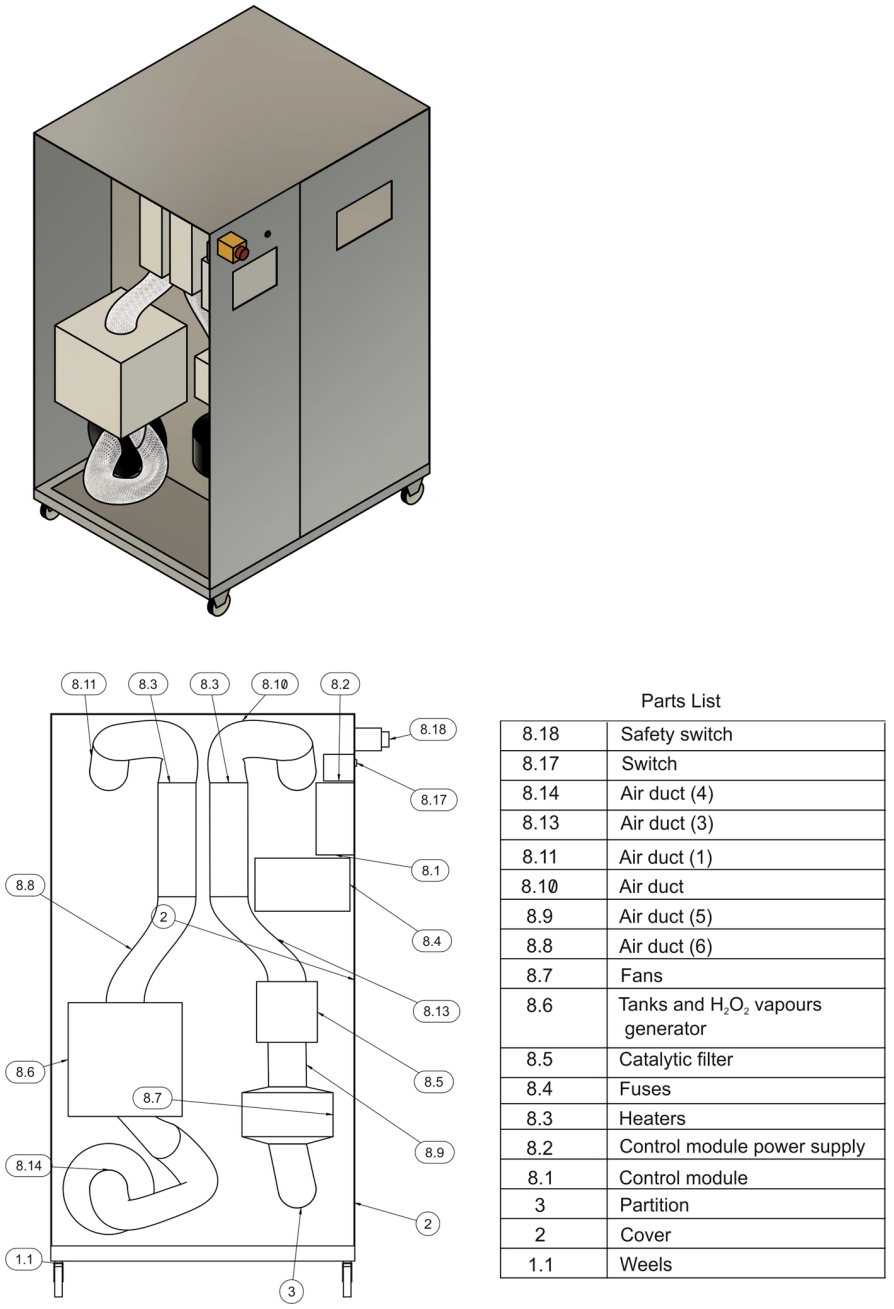
Schematic diagram of a mobile sterilization station (utility model pending application No. W.130269 [WIPO ST 10/C PL130269U]).

The device was made of 0.2-mm-thick stainless steel, which provided the structure with high resistance to corrosion and chemical agents (steel type: 1.4301/1.4404). The body of the device was mounted on a steel frame. The prototype was equipped with elements to facilitate transport, i.e., a wheeled chassis with four lockable swivel wheels. The prototype was built by Tungsten Inert Gas (TIG) welding. The welds and elements of the prototype body were ground and polished. The sterilization chamber (working chamber) contained two UV-C lamps with a total power of 36 W. Additional movable elements enabled sterilization of the entire arrangement (insertable and removable shelves, sterilization baskets, drainers, etc.). The device integrated proprietary solutions in the form of a cover for UV-C radiators, a container for the hydrogen peroxide solution, an ultrasonic vapor generator, and frames for mounting catalytic filters (utility model pending application No. W.130269 [WIPO ST 10/C PL130269U]). The control module was placed in the front portion of the device and was equipped with a touch screen. The application controlling the device function works in two modes: (1) user mode, where the START and STOP buttons appear, as well as the sterilization time progress bar, and (2) service mode, which allows the working times of the UV-C radiators and H_2_O_2_ vapor generator to be modified. In addition, this application has a closed-door sensor, which prevents the device from initiating operation when the door is open (Fig. 4).

### Optimization of sterilization conditions

#### *H*_*2*_*O*_*2*_* concentration and temperature measurements*

The H_2_O_2_ concentration and temperature were monitored inside the working chamber of the mobile sterilization station using PEROXCAP HPP272 transmitters to measure the hydrogen peroxide vapors, humidity, and temperature (Vaisala Insight software, version 1.0.2.76, Vaisala Oyj, Vantaa, Finland). When using the designed system, it was possible to control the sterilization process in terms of the concentration of the sterilizing agent, i.e., H_2_O_2_ vapor and temperature.

#### Microorganisms used for sterilization quality tests

*The Geobacillus stearothermophilus* (ATCC 7953) is the indicating bacterial spore-forming strain of safe microorganism in commercially available standardized test that may be used for laboratory purposes but also by non-specialized users.

*Bacillus subtilis* strain (ATCC 6051) represents gram-positive and spore forming bacteria, *Escherichia coli* (ATCC 13706) represents gram-negative bacteria and *Candida albicans strain (*ATCC 3147) represents yeast. All used microorganisms are common in the environment and may occur i.a. on human skin.

#### Growth media used in tests

Growth of microorganisms was determined using culture media such as:

Tryptone Soy Broth medium (TSB; 1.5% casein peptone, 0.5% sodium chloride, 0.5% soy peptone; pH 7.3; Merck, Darmstadt, Germany) for cultivation of tested bacterial strains in liquid culture.

Luria–Bertani Agar medium (LA; 1% tryptone, 1% sodium chloride, 0.5% yeast extract; 1.5% agar; pH 6.8–7.2; Merck) for cultivation and enumeration of total bacteria on solid medium.

Sabouraud agar (1% peptone, 4% dextrose, 1.5% agar; pH 5.6; BTL Sp. z o. o.) for fungi, yeasts and molds cultivation and enumeration on solid medium.

#### Commercial sterilization quality controls

Standardized biological quality control samples, compliant with the ISO 11138-1^15^ standard, containing *Geobacillus stearothermophilus* (ATCC 7953 spores; CFU = 1.3 × 10^5^; BIONOVA Terragene, Santa Fe, Argentina) and a liquid culture medium with a color indicator were used. Briefly, on two levels (upper and lower) created using mesh shelves there were placed three doublets of vials. After sterilization process internal capsule filled with culture media supplemented with indicator was squeezed to release the liquid into the vial containing microbial spores. The vials were incubated for 24 h at 60 °C. Purple color in the medium after the incubation period indicated that the microorganisms were killed, whereas yellow medium indicated that they survived and underwent cell division, i.e., the sterilization was not successful. As a positive control vial without sterilization was used as described above. Moreover, control vials contained chemical label with indicator changing color when exposed to H_2_O_2_ vapors; a violet color indicated that the vial was not exposed to H_2_O_2_, and a green indicated exposure to H_2_O_2_ vapors. This test helped to assess the minimum time and concentration of H_2_O_2_ vapors for successful sterilization process.

Additionally, the effectiveness of the H_2_O_2_ vaporization technique was monitored using two types of commercial chemical tests: Chemdye CD40 type-4 (ISO 11140-1^16^; Terragene) and Excelsior Hydrogen Peroxide (VH2O2) Process Indicator Strips type-1 (ISO 11140-1^16^; Excelsior Scientific, Cambridgeshire, UK). In both cases, the test field changing to a green color confirmed proper H_2_O_2_ vaporization. About 20 test strips were placed in different locations of the chamber to evenly cover most of the chamber space.

#### Microbiological tests

The sterilization process was evaluated against various systems, i.e., exposing *Bacillus subtilis* spores and *Escherichia coli* cells to the sterilizing agent for various exposition time (up to 60 min), H_2_O_2_ vapor concentrations (50–150 ppm), and temperatures (20–40 °C). Test where prepared on Whatman’s paper discs (5 mm diameter) containing 2 × 10^6^ CFUs of *B. subtilis* spores or 2 × 10^7^ CFUs of *E. coli* cells. Dry discs were used in sterilization processes and after completion of the cycle the discs were floated with PBS buffer and vortexed to suspend bacterial cells in the buffer. As a control, discs without sterilization exposure were used. 100 µL of PBS with suspend microorganisms were transferred into TSB medium and incubated for 72 h at 37 °C. During the incubation the growth of microorganisms in the medium was monitored. As a negative control, disc without sterilization cycle was used. Moreover, assessing the sterilization capacity of the device, cellulose-cotton discs (diameter = 1 cm, thickness = 2–4 mm) were soaked with the microbial cultures such as *E. coli*, *B. subtillis* and *C. albicans* and subjected to the sterilization process in the working chamber. About 20–30 disks were placed in different location of the chamber to evenly cover most of the chamber space. After completing the sterilization process, the microorganisms were rinsed from the discs with PBS (1 mL) and seeded on Petri dishes with LA or Sabouraud medium. All tests were performed in triplicate. Additionally, flow cytometric analyses of the microorganisms’ viability were performed (for details, see “Flow cytometry analysis” section).

#### Flow cytometry analysis

**Figure 5:**
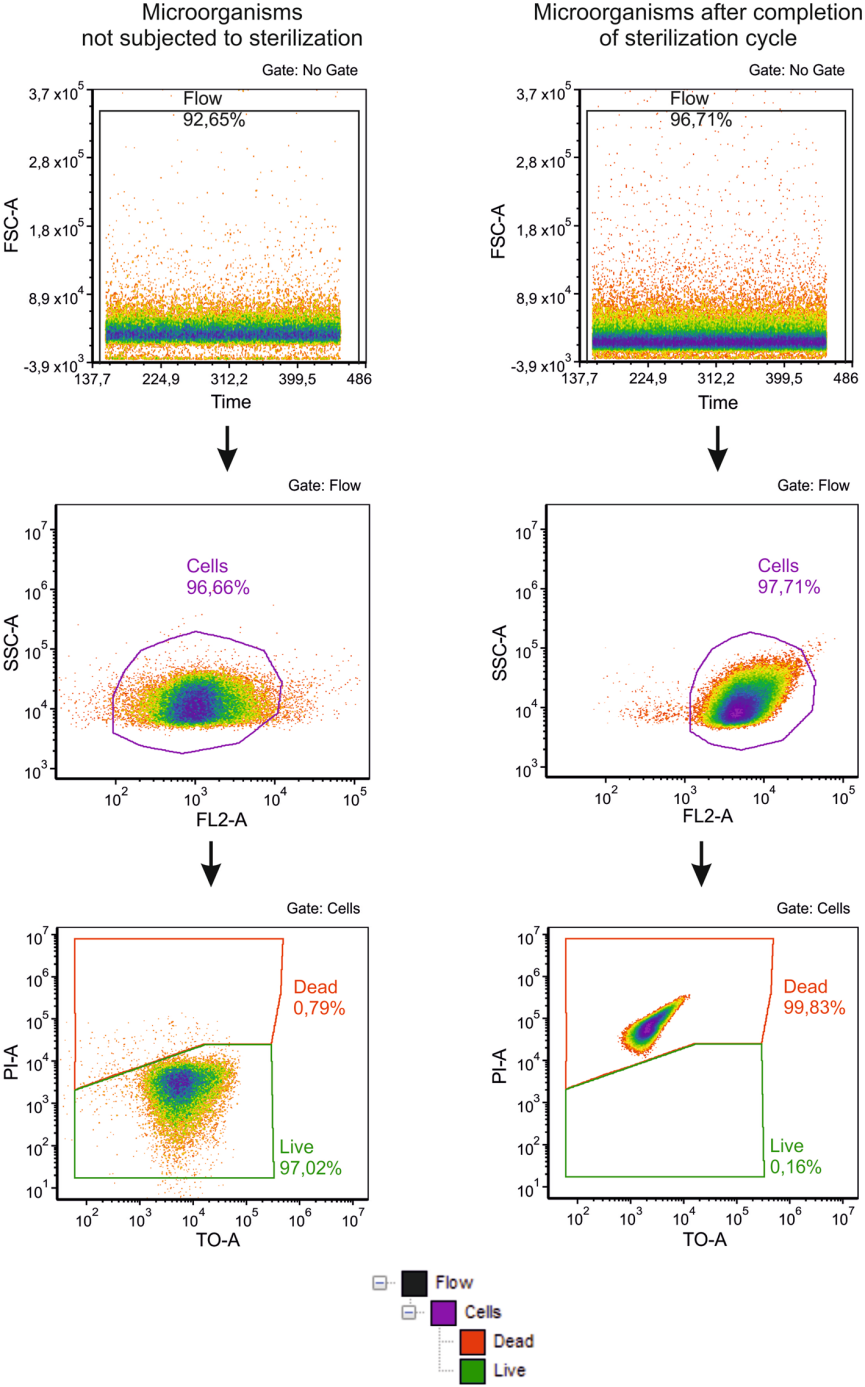
Gating strategy for flow cytometric analyses.

In addition to the classic microbiological tests, the flow cytometry technique was used. PBS with suspended microorganisms extracted from Whatman’s paper discs coated with tested bacterial strains (as described above) was used in flow cytometry assay. As a control, discs without sterilization exposure were used. Such prepared samples and controls were used in flow cytometry analysis to assess microbial viability, as well as proportion between live and dead cells, using BD Cell Viability Kits (BD Biosciences, San Jose, CA, USA). Briefly, 5 µL of dye solution containing thiazole orange (TO) and propidium iodide (PI) were added to 500 µL of cell solution. The samples were vortexed and incubated for 5 min in the dark at room temperature. The data was acquired on a flow cytometer BD Accuri^TM^ C6 Flow Cytometer (Becton Dickinson, Franklin Lakes, NJ, USA) for 30 s at the fast flow rate (66 μL/min) with an SSC-H threshold of 10,000 to exclude debris. To validate the test, viable and artificially killed analyzed microorganisms were used. The results were calculated using BD Accuri™ C6 (version 1.0.264.21) and FCS Express (version 4.07.0020 RUO Edition; De Novo Software, Los Angeles, CA, USA) software programs. Gating strategy is presented in Fig. 5.

#### Sterilizing air or material surfaces in the working chamber using full sterilization cycles

The air from the prototype was controlled using a MicroBio MB1 Bioaerosol Sampler (Cantium Scientific Ltd., Dartford, UK), placed in the center of the mobile sterilization station working chamber before and after sterilization process (0, 5, 10, 15, or 20 min). Tests were performed as follow: 10 dm^3^ of air from the mobile sterilization station was directed onto Petri dishes with the LA and Sabouraud agar media (Merck) to determine total bacteria on LA agar medium as well as fungi and molds on Sabouraud agar present in the air, respectively. After, exposition both media were incubated for 48 h at 37 °C followed by counting of the colony forming units (CFUs) per 1 m^3^ of air and calculated, according to Eq. (1),

1 $$CFU = \frac{L1 + L2 + S2 + S2}{4} \times 100$$

where L1 and L2 are the number of total bacterial colonies grown on LA agar medium and S1 and S2 are the number of fungi and molds colonies grown on the the Sabouraud medium.

Microbiological testing was completed by placing Petri dishes with LA or Sabouraud agar medium containing freshly-spread cultures of bacteria *B. subtilis* (10^9^ CFU/mL) and yeast *Candida albicans* (10^7^ CFU/mL), respectively, in the sterilization station’s working chamber. After sterilization process plates were closed immediately and incubated for 72 h at 37 °C, at which time-point the growth of seeded microorganisms was evaluated.

### Focused research and testing under real conditions

Focused research and testing under real conditions was conducted with participation of volunteers (focus group and members of the state fire brigade, respectively) who operated the sterilization station and then answered the questionnaire. All testing procedures, including those requiring participation of volunteers approved by the Local Ethics Committee at the Regional Medical Chamber in Szczecin (approval no. 05/KB/VII/2019), were approved by the “Socially responsible Proto_lab” project board under the West Pomeranian voivodeship and carried out in accordance with relevant guidelines and regulations. Participants were fully informed of the experimental procedures before giving their consent to participate.

## Data Availability

The datasets generated during and/or analyzed during the current study are available from the corresponding author on reasonable request.
